# Interventions to Dispel Neuromyths in Educational Settings—A Review

**DOI:** 10.3389/fpsyg.2021.719692

**Published:** 2021-10-13

**Authors:** Luc Rousseau

**Affiliations:** Department of Psychology, Laurentian University, Greater Sudbury, ON, Canada

**Keywords:** education, neuromyths, interventions, false beliefs, refutation texts, teaching practices

## Abstract

*Neuromyths* are misconceptions about the brain and learning, for instance *Tailoring instruction to students' preferred “learning styles” (e.g., visual, auditory, kinesthetic) promotes learning*. Recent reviews indicate that the high prevalence of beliefs in neuromyths among educators did not decline over the past decade. Potential adverse effects of neuromyth beliefs on teaching practices prompted researchers to develop interventions to dispel these misconceptions in educational settings. This paper provides a critical review of current intervention approaches. The following questions are examined: Does neuroscience training protect against neuromyths? Are refutation-based interventions effective at dispelling neuromyths, and are corrective effects enduring in time? Why refutation-based interventions are not enough? Do reduced beliefs in neuromyths translate in the adoption of more evidence-based teaching practices? Are teacher professional development workshops and seminars on the neuroscience of learning effective at instilling neuroscience in the classroom? Challenges, issues, controversies, and research gaps in the field are highlighted, notably the so-called “backfire effect,” the social desirability bias, and the powerful intuitive thinking mode. Future directions are outlined.

## Introduction

*Neuromyths* are misconceptions about the brain and learning. Among the most prevalent neuromyths are the two following assertions: *Tailoring instruction to students' preferred “learning style” (e.g., visual, auditory, kinesthetic) promotes learning*; and *People are either “left-brained” or “right-brained,” which helps to explain individual differences in learning*. The most pervasive neuromyths contain a “kernel of truth” (Grospietsch and Mayer, 2018). On the one hand, classical neuroscience research did provide solid basic evidence that sensory inputs are processed by modality-specific cerebral areas (Penfield and Boldrey, 1937), and that the human brain displays a certain degree of functional hemispheric lateralization (Gazzaniga et al., 1962, 1963). On the other hand, though, the idea of a “dominant” sensory modality or cerebral hemisphere is not supported by neuroscience (Calvert et al., 2000; Nielsen et al., 2013; Pasqualotto et al., 2016). Due to fatal mutations from kernels of truth, neuromyths are typically defined as distortions, oversimplifications, or abusive extrapolations of well-established neuroscientific facts (Organisation for Economic Co-operation and Development, 2002; Pasquinelli, 2012; Howard-Jones, 2014).

### Prevalence and Persistence of Neuromyth Beliefs Among Educators

To measure neuromyth endorsement, Dekker et al. (2012) devised a questionnaire containing a mix of 15 statements considered as neuromyths (e.g., *We only use 10% of our brain*) and 17 statements considered as factual general knowledge about the brain and learning, also known as “neuro-facts” or “neuro-hits” (e.g., *Learning occurs through modification of the brain's neural connections*). Despite the lack of a robust factorial structure underlying Dekker et al.'s 2012 neuromyth sub-scale (Horvath et al., 2018), in the last decade, numerous surveys adapted from this questionnaire have been conducted in educational settings in more than 20 countries around the world. A recent review (Torrijos-Muelas et al., 2021), which targeted 24 articles published in the years 2012–2020, indicates that the prevalence of beliefs in neuromyths among educators, ranging from 27.3 to 84.5% (weighted average = 52.1%)^1^, shows a remarkably stable trend. The authors found no evidence of decline over the eight-year period, suggesting strong persistence of neuromyth beliefs among educators. With regards to the most prevalent, VAK (i.e., visual, auditory, kinesthetic) “learning styles” neuromyth, another recent review (Newton and Salvi, 2020), which targeted 33 articles (containing a total of 37 samples) published in the years 2009–2020, indicates a high prevalence of beliefs in this neuromyth among educators, ranging from 58 to 97.6% (weighted average prevalence = 89.1%), with no evidence of decline over the eleven-year period.

The strong persistence of neuromyth beliefs in educational settings is surprising, in light of a host of studies, conducted in the same time period, that failed to find evidence for positive learning outcomes gained from matching instruction to “learning style” preferences (Krätzig and Arbuthnott, 2006; Rogowsky et al., 2015, 2020; Knoll et al., 2017; Cuevas and Dawson, 2018; Husmann and O'Loughlin, 2019; for reviews, see Pashler et al., 2008; Aslaksen and Lorås, 2018; Rousseau et al., 2018), from matching instruction to *multiple intelligences* profiles (Ferrero et al., 2021; Rousseau, 2021), or from applying the Brain Gym® methods (Hyatt, 2007; Stephenson, 2009; Spaulding et al., 2010; Watson and Kelso, 2014; Cancela et al., 2015; Kroeze et al., 2016), and despite neuromyth “busting” efforts through national newspapers (e.g., Hood et al., 2017), professional journals in education (e.g., Willingham, 2005, 2006; Blanchette Sarrasin and Masson, 2015), Internet blogs/general audience electronic media (e.g., Rousseau, 2020), and popular books (e.g., Adey and Dillon, 2012; De Bruyckere et al., 2015).

### Harmful Effects of Neuromyths

The primary motivation to survey neuromyth beliefs in educational settings was the assumption that endorsing neuromyths has adverse effects on teaching practices. For instance, in their seminal study, Dekker et al. (2012) warned that “[…] it would be of concern if neuromyths were found in this sample, because these teachers will be most eager to implement (wrong) brain-based ideas in educational practice” (p. 1). Although the high prevalence of neuromyth beliefs in educational settings is, in itself, a threat to the “evidence-based” movement, in education (Davies, 1999; Wiseman, 2010), there is still no supporting data for a causal chain from educators' beliefs in neuromyths to adoption of poor teaching practices and/or negative impact on students' academic achievement (Hughes et al., 2021). That beliefs in neuromyths make for bad teaching has been challenged by Horvath et al. (2018). They found that the prevalence of neuromyth beliefs among a group of internationally renowned, award-winning teachers, was nearly identical to that reported in the literature among non-award-winning teachers. As acknowledged by the authors themselves, this finding provides indirect, not causal evidence for the irrelevance of neuromyth beliefs to teacher effectiveness. In a similar vein, Krammer et al. (2021) recently reported that endorsing/rejecting neuromyth survey statements had no impact on pre-service teachers' academic grades. On the surface, this finding suggests that believing in neuromyths is irrelevant to prospective teacher effectiveness. But underneath the surface, it may suggest that neuromyths coexist with valid scientific knowledge in teacher students' minds, the latter being selectively used in academic assignments and exams. That prospective teachers avoid using neuromyths in academia, though, does not preclude their use in their future teaching practice.

Direct evidence of the harmful effects of neuromyth beliefs on instruction might still be lacking, but educational policies are already influenced by neuromyths, which may lead to unreasonable spending of resources and money. For instance, in the US, aspiring teachers in 29 states and the District of Columbia are required to pass licensing exams whose free, state-provided study materials reference students' learning styles, and (except for Massachusetts) clearly advocate for relevance or application of learning style theory (Furey, 2020). In the UK, the “Most learning happens in the first 3 years” neuromyth has been evoked to support nursery schools subsidized from public funds (Blakemore and Stern, 2005). And based on the false belief that listening to Mozart music boosts IQ, a pervasive neuromyth (Latendresse et al., 2006), in 1998, the state of Florida has passed Senate Bill No. 660 for day-care centers to play classical music to infants and toddlers, and the Georgia Governor asked for $105,000 for the production and distribution of classical music to newborns (Pasquinelli, 2012). Given such a situation, efforts at dispelling neuromyth beliefs in educational settings are worthwhile.

In a recent review of the current state of research on neuromyths, Grospietsch and Lins (2021) indicate that “there is too little work on developing and evaluating intervention approaches to combat neuromyths” (p. 1). The present critical review aims at providing a synthesis of current intervention approaches, as well as highlighting challenges, issues, controversies, and research gaps in the field.

## Does Neuroscience Training Protect Against Neuromyths?

Because neuromyths are defined as abusive extrapolations/oversimplifications of legitimate neuroscience knowledge, it is not surprising that neuroscience training is, by far, the primary recommendation made by authors of neuromyth surveys to dispel them. In many published surveys, authors report the correlation found between endorsement of general knowledge statements about the brain and learning (“neuro-hits”) and endorsement of neuromyth statements. Quite remarkably, the direction of the correlation depends upon the sample composition. This is probably the most robust pattern to be found in the literature on neuromyths. When the sample is composed of in-service teachers, the trend is for the neuromyth score to be *positively* correlated with the neuro-hit score (Dekker et al., 2012; Gleichgerrcht et al., 2015; Ferrero et al., 2016; Hughes et al., 2020; Tovazzi et al., 2020; Bissessar and Youssef, 2021). By contrast, when the sample is composed of pre-service teachers, the trend is for the two scores to be *negatively* correlated (Howard-Jones et al., 2009; Papadatou-Pastou et al., 2017; Carter et al., 2020,^2^; Ching et al., 2020; but see Kim and Sankey, 2018, for an exception^3^). In other words, the more valid general knowledge about the brain and learning is held, the *more* neuromyths are endorsed by in-service teachers, but the *less* neuromyths are endorsed by pre-service teachers. The first trend is puzzling. Dekker et al. (2012) speculated that compared to prospective teachers, in-service teachers are likely to have been exposed to more sources of information—both correct and incorrect—about the neuroscience of learning, and due to their eagerness to implement neuroscience into their teaching practice, coupled with their lack of expertise in neuroscience, it is difficult for them to differentiate between this correct and incorrect information. The second trend is encouraging, as it suggests that general knowledge about the brain could provide teacher students with a “protective shield” against neuromyths (Papadatou-Pastou et al., 2017), and that incorporating neuroscience training into initial teacher education may potentially help to dispel neuromyths. But given the correlational nature of the reported relationship, only pre/post experimental designs could unveil a causal link between more neuroscience knowledge and less neuromyth endorsement.

Using a pre/post experimental design, Im et al. (2018) examined the effect of taking a course in educational psychology on neuromyth endorsement in a sample of pre-service teachers. The course textbook included a section addressing unaccredited “brain-based” teaching approaches and debunking two major neuromyths. Results showed no reduction in endorsement of neuromyth survey statements at the end of the semester, in both the experimental sample and a control sample of peers from the same cohort who did not take the course. In turn, taking the educational psychology course increased neuroscience knowledge, as more neuro-hit survey statements were endorsed by prospective teachers in the experimental group.

Converging evidence supports the view that the mere exposure to neuroscience training is insufficient to dispel neuromyths. Kowalski and Taylor (2017) have shown that first-year undergrad students stick to their psychological misconceptions after taking an Introduction to Psychology course covering scientific knowledge conflicting with such misconceptions. Similarly, Grospietsch and Mayer (2018) found that pre-service biology teachers did not show a decline in neuromyth beliefs after being enrolled into a cognitive neuroscience course. It could be argued that taking one single neuroscience course is insufficient for a protective effect to emerge. Actually, Macdonald et al. (2017) found that “high exposure” to neuroscience during academic training (having taken several courses related to the brain or neuroscience courses at the college/university level) significantly predicted a lower rate of neuromyth endorsement in educators, but the effect size was qualified by the authors as “modest” ($\eta_{\text{p}}^{2}$ = 0.035). In fact, educators who took several neuroscience courses endorsed, on average, only 1.3 less neuromyths (over 7) than educators who took no such course at all.

According to Grospietsch and Mayer (2018), because neuromyths are deeply rooted in personal belief systems, the mere exposure to a neuroscience course only implicitly confronts neuromyths to neuroscience knowledge. To effectively dispel neuromyths, *explicitly* stating how neuroscience knowledge contradicts them is a key factor. This is the central assumption underlying refutation-based interventions.

## Are Refutation-Based Interventions Effective at Dispelling Neuromyths, and Are Corrective Effects Enduring in Time?

Based on the literature on conceptual change, the intervention approach developed by Grospietsch and Mayer (2018) aimed at creating a *cognitive conflict* between neuromyth beliefs and neuroscience knowledge. Participants were pre-service biology teachers enrolled in a cognitive neuroscience course. Several times during the semester, prospective teachers engaged in personal, written reflections. Participants in the experimental group reflected upon a given neuromyth statement, both before and after reading a refutation text *explicitly* stating how neuroscience concepts seen in class contradict false claims made in the neuromyth statement. Critically, the refutation text addressed both legitimate neuroscience knowledge (the “kernel of truth”) and abusive extrapolations/oversimplifications embedded in the neuromyth statement. Participants in the control group simply reflected upon their own personal learning in the course, not on neuromyth statements. That some neuroscience concepts seen in class contradict false claims made in neuromyth statements was only *implicit* in this group. Consonant with Im et al.'s 2018 previous findings, control participants did not show a decline in the endorsement of 11 common neuromyths after being merely exposed to the neuroscience course. By contrast, in the experimental group, the average endorsement of the seven neuromyths that were addressed in the conceptual change sessions dramatically decreased from 74 (pre-test) to 22% (post-test). The effect spilled over four non-target neuromyths that were only indirectly addressed in the conceptual change sessions. It remains unclear, though, whether refutation texts alone, personal reflections upon neuromyth statements alone, or a combination of both (the conceptual change component), underlay efficiency at reducing neuromyth beliefs in this study. In addition, whether the corrective effect was enduring is unknown, as no follow-up evaluation was conducted by Grospietsch and Mayer (2018).

Interestingly, a similar pattern of results was obtained by Kowalski and Taylor (2017) in a classroom-based intervention to dispel psychological misconceptions (Lilienfeld et al., 2010). Note that there is considerable overlap^4^ between some of these psychological misconceptions (e.g., *Most people use only 10% of their brain power*; *Playing Mozart's music to infants boosts their intelligence*; *Some people are left-brained, others are right-brained*; *The defining feature of dyslexia is reversing letters*) and neuromyths. First-year Psychology students were first asked to fill out a survey measuring endorsement of 25 misconceptions, before attending one of eight sections of an Introduction to Psychology course. Eight misconceptions taken from the survey were targeted for the intervention. In one section of the Introduction to Psychology course, three target misconceptions (including the “learning styles” and the “Mozart music” neuromyths) were explicitly mentioned in the classroom, followed by a refutation lecture (the correct conception was taught), a reading, and a discussion. In another section of the course, the correct conceptions were taught, followed by a reading, but the three misconceptions (including the “first 3 years” neuromyth) were not explicitly mentioned (standard lecture). In yet another section, the two other target misconceptions were not explicitly mentioned nor was any correct information provided (control condition). Refutation lectures were shown to be more effective than standard lectures (i.e., mere exposure to correct information) to dispel psychological misconceptions. Impressively, the refutation-based corrective effect, evidenced at the end of the Introduction to Psychology course, was maintained at the first follow-up evaluation (16 weeks later), as well as at the second one (2 years later).

Menz et al. (2021c) replicated Kowalski and Taylor's 2017 study, this time in a sample of pre-service teachers enrolled in an educational psychology course. A different set of psychological misconceptions (again including neuromyths) was used. Contrary to previous findings in first-year Psychology students, Menz et al. (2021c) found that both the standard and the refutation lectures were efficient at reducing endorsement of target misconceptions. For the refutation lecture condition, the corrective effect spilled over the misconceptions that were not addressed in the lectures, while for the standard lecture condition, the corrective effect was limited to target misconceptions. Although both conditions produced a corrective effect, refutation lectures were more effective than standard ones. Gains were maintained at the 6-month follow-up evaluation for the refutation lecture condition, but the spill over vanished. For the standard lecture condition, gains were not enduring.

In the field, not all refutation-based pre-/post-experimental designs are embedded into an academic course. A simpler format proceeds in three steps: (Step 1) administering participants a neuromyth survey adapted or extended from Dekker et al. (2012); (Step 2) having participants read a *refutation text*, i.e., scientific evidence against target neuromyths; and (Step 3) re-administering the same neuromyth survey as in Step 1. The refutation text, couched in plain language, specifies how the claim made in the neuromyth statement (e.g., *Short bouts of co-ordination exercises can improve integration of left and right hemispheric brain function*) conflicts with knowledge about the functioning of the brain (e.g., *The left and right sides of the brain are massively connected through a bundle of 200–300 million neural fibers called the corpus callosum*) and/or synthesizes one to many lines of research showing that the claim does not withstand empirical scrutiny. Most studies include a follow-up (Step 4) that consists in administering the same neuromyth survey as in Step 1 and 3.

Ferrero et al. (2020a) first measured endorsement of 18 neuromyths and 18 neuro-hit survey statements (presented in random order) in a sample of in-service teachers (Step 1). Forty-five days later, participants read refutation texts (Step 2). Nine of the 18 neuromyths were targeted for the intervention, based on their endorsement level (3 strongly endorsed, 3 intermediate, 3 weakly endorsed) in a pilot study. At each endorsement level, there were three conditions, orthogonally assigned to the three target neuromyths: a text providing information about the origin of the neuromyth and refuting it (TO for Text + Origin); a text refuting the neuromyth without information on its origin (TA for Text Alone); and no refutation nor information about the origin (NT for No Text—the control condition). Right after reading the six refutation texts, participants were re-administered the neuromyth survey (Step 3). Finally, 30 days later, the neuromyth survey was again administered (Step 4—follow-up evaluation). For both experimental conditions (TO and TA), Ferrero et al. (2020a) observed a “V” result pattern, i.e., a significant drop in neuromyth endorsement from the pre-intervention baseline level (Step 1) to the post-intervention evaluation (Step 3), followed by a significant rise, almost at the baseline level, 30 days later (Step 4—follow-up evaluation). The authors concluded that the effectiveness of refutation texts to dispel neuromyth beliefs was short-lived. Interestingly, Ferrero et al. (2020a) also measured intentions to use neuromyth-derived teaching practices at Step 3 and 4, and observed a significant rise from Step 3 to 4. The authors interpreted this rise as a “backfire effect,” i.e., the amplification of people's personal convictions when confronted with counterevidence (Nyhan and Reifler, 2010). However, because the baseline level (Step 1) was unknown for intentions to use neuromyth-derived teaching practices, there was no way to assess whether intentions actually backfired. A clear backfire effect would be evidenced by intentions rising *over* the baseline level after the intervention. Ferrero et al. (2020b) conducted the same intervention in a sample of pre-service teachers, this time assessing both beliefs and intentions at Step 1, 3, and 4. A similar “V” pattern was observed for both beliefs and intentions. As was observed for beliefs, at Step 4, intentions almost returned to the baseline level (Step 1), ruling out a “backfire effect” as a viable explanation of the findings.

An alternative interpretation was suggested by Newton and Salvi (2020). When participants respond in the way they perceive the study outcome “desired” by the researchers, they are said to succumb to the *social desirability bias* (Nederhof, 1985). Because neuromyth statements are explicitly referred to in the refutation texts, participants could easily figure out, when filling out the same neuromyth survey again, that researchers intended to prove that the refutation texts were efficient at debunking misconceptions. Participants may have tried to fulfill researchers' “desires” by providing lower endorsement ratings to neuromyth statements after reading the refutation texts, rather than base their responses on their own judgment. As the authors reasoned for refutation-based interventions aimed at dispelling the “learning styles” neuromyth:

It seems reasonable to conclude that there is a risk of social desirability bias in these studies; if participants have been given training which explains the lack of evidence to support Learning Styles, then they might be reasonably expected to disagree with a statement which supports matching [instruction to Learning Styles] (Newton and Salvi, 2020, p. 12).

Actually, any drop in beliefs or intentions from Step 1 to Step 3 (e.g., Newton and Miah, 2017; Grospietsch and Mayer, 2018; Menz et al., 2021a,b,c) could be interpreted as reflecting a social desirability bias (Newton and Salvi, 2020). A social desirability bias could also account for the “V” pattern observed by Ferrero et al. (2020a,b) by assuming the bias to peak right after reading the refutation text (Step 3), but dissipating as time goes by (Step 4—follow-up evaluation, 30 days later).

In contrast to Ferrero et al. (2020a,b), Lithander et al.'s 2021 refutation texts were quite simple. Whereas, Ferrero et al. (2020a,b) refutation texts contained, on average, 180.66 words for the origin of the neuromyth and 149.55 words for the refutation *per se*, Lithander et al. (2021) used substantially shorter texts. There were four different conditions: (1) the Refutation Only condition (the neuromyth statement, followed by “This statement is false”), (2) the Refutation + Explanation condition (the explanation held in one to three sentences containing, on average, 56.2 words), (3) the Refutation + Explanation + Image condition (the image, from a classroom to a fMRI picture, was associated with the explanation), and (4) No Refutation. The authors hypothesized that the Refutation + Explanation condition would be more effective than the Refutation Only condition but, quite surprisingly, the Refutation Only Condition (“This statement is false”) was as efficient as the two other conditions at reducing neuromyth beliefs, with similar enduring effects at both follow-up evaluations (1 week and 1 month later). This unpredicted finding is at odds with Grospietsch and Mayer's 2018 assumption—based on the conceptual change literature—that neuromyths should be confronted with legitimate neuroscience knowledge to be altered. At this point, Lithander et al.'s 2021 “simple refutation” finding remains highly puzzling. Of note, in Ferrero et al.'s studies 2020a; 2020b, the more elaborate TO (Text + Origin) condition did not lead to a larger effect than the simpler, TA (Text Alone) condition, also an unpredicted finding. The simplicity bias (Chater and Vitányi, 2003; Lombrozo, 2007; Feldman, 2016) may possibly be at play here. Because people prefer simple to complex explanations, simple, brief rebuttals might show to be more effective than refutation texts “overkilling” misconceptions with several counterarguments (Lewandowsky et al., 2012).

Unlike Ferrero et al. (2020a,b), most studies (Kowalski and Taylor, 2017; Lithander et al., 2021; Menz et al., 2021c) observed that the refutation-based corrective effects were maintained at the follow-up evaluation. Such a discrepancy in outcomes could be attributed to differences in sample composition, in the selection of target neuromyths, in the intervention format (refutation lectures vs. refutation texts), or in the time period between Step 3 and 4. In Lithander et al.'s 2021 studies, conducted in the general population as well as in first-year Psychology students, the drop in neuromyth endorsement was still present at the first (1 week later) as well as at the second follow-up evaluation (1 month later). Although these findings may suggest that non-educators are less resistant to educational neuromyths (Lithander et al., 2021), Menz et al. (2021c) conducted their study in pre-service teachers and observed enduring gains (reduced beliefs) at a 6-month follow-up evaluation. More research will be needed to identify critical variables at play here.

## Why Refutation-Based Interventions Are Not Enough: The Powerful Intuitive Thinking Mode

According to *dual processing* theories of cognition (Kahneman, 2011; Evans and Stanovich, 2013), people engage in two modes of thinking. Type 1 thinking is a fast, automatic, and intuitive mode that relies on anecdotal evidence and personal experience, while Type 2 thinking is a slower, effortful, and reflective mode that relies on reason and objective analysis. Much interestingly, Bensley et al. (2014) found that participants who achieved high scores on critical thinking skills tests endorsed fewer psychological misconceptions, while by contrast, participants who achieved high scores on the Faith of Intuition scale endorsed more. These findings suggest that effective interventions to dispel neuromyths should focus not only on activating Type 2, rational thinking (refutation-based interventions), but also on mitigating Type 1, intuitive thinking (Bensley and Lilienfeld, 2017).

Newton and Miah's 2017 study provides a clear indication that neuromyths could still prevail^5^, through intuitive thinking, in educators' minds, despite a seemingly efficient refutation-based intervention focused on rational thinking. They first reported that 64% of teachers in higher education agreed with Dekker et al.'s 2012 “learning styles” neuromyth statement [*Individuals learn better when they receive information in their preferred “learning style” (e.g., auditory, visual, kinesthetic*)]. Then, respondents were presented with scientific evidence showing that learning styles are not effective. After the refutation-based intervention, 90% of respondents agreed that “The theory of Learning Styles is conceptually flawed—it does not account for the complexity of understanding” (i.e., endorsement of the “learning styles” neuromyth dropped from 64 to 10%). But quite intriguingly, among those, 31.6% agreed with the following statement: “In light of the information presented, I plan to try and account for individual student Learning Styles in my teaching.” In other words, nearly one third of the respondents entertained, simultaneously, the neuromyth and legitimate neuroscience knowledge contradicting it. Newton and Miah (2017) speculated that a social desirability bias could account for respondents' agreement with the scientific statement about learning style theories being conceptually flawed. But why nearly one third of the respondents stuck to the “learning styles” neuromyth is suggested by another finding. Eighty-nine percent of those who indicated, after the intervention, that they were still planning to try and account for learning styles in the classroom, agreed with the following statement: “Even though there is no ‘evidence base’ to support the use of Learning Styles, it is my experience that their use in my teaching benefits student learning.” In other words, in this sub-sample of educators, intuitive thinking prevailed over rational thinking.

Past research has found that teachers rely on various accredited and unaccredited sources for information about neuroscience and education (e.g., Zambo and Zambo, 2009). Teachers also came across so-called “brain-based” approaches through their schools, other teachers, trainers, electronic media and conferences (Simmonds, 2014). Because teachers' access to peer-reviewed scientific papers is relatively limited (Dekker et al., 2012), anecdotal sources of evidence, in the form of narratives from colleagues or personal experience (e.g., observations in the classroom), may take over scientific sources to support held misconceptions, through the *confirmation bias* (Riener and Willingham, 2010). It could be argued that refutation texts, based on scientific evidence, do not compete well with such powerful anecdotal evidence. As speculated by Pasquinelli (2012), the availability and familiarity cognitive biases could play a critical role here: “The teacher who has adopted Brain Gym® methods is ready to deliver an emotionally rich story far more memorable than the negative statistics drawn from meta-analyses” (p. 93).

Blanchette Sarrasin et al. (2019) and Menz et al. (2021b) found that teachers are more likely to rely on intuitive/anecdotal sources of evidence than on rational/scientific ones to support their level of agreement with neuromyth survey statements. In both studies, after each response, participants were asked to indicate which sources they based their response on (more than one source could be checked). Blanchette Sarrasin et al. (2019) suggested five main sources to their participants. To support their agreement with neuromyth statements, intuitive/anecdotal sources of evidence [intuition (*It makes sense to me)*; narratives from other people (colleagues; principals/counselors; family/friends); personal experience (*I notice it in my practice*)] were disproportionately checked by in-service teachers, in comparison to scientific sources of evidence (books and textbooks; popular articles; scientific papers), except for the “10%” neuromyth. Menz et al. (2021b) suggested six main sources to their participants. To support their agreement with psychological misconceptions, pre-service teachers checked anecdotal sources of evidence (narratives from other people; personal experience) significantly more often than scientific sources of evidence (lectures; scientific research).

Remarkably, Menz et al. (2021b) found that the sub-sample of pre-service teachers who indicated anecdotal evidence as the primary source of their agreement with neuromyth statements not only endorsed them to a greater extent than the sub-sample who indicated scientific evidence as their primary source of agreement, they *also* showed a weaker reduction in neuromyth beliefs after reading refutation texts. On that basis, Menz et al. (2021a) reasoned that if anecdotal evidence is more powerful than scientific evidence, then refutation texts based on anecdotal evidence (narratives from other people) may be more effective than standard, science-based refutation texts, to dispel neuromyths. In their study, refutation texts were identical, except for the referenced source (*The current state of research in educational psychology indicates that […]*, vs. *Companioned teachers tell you that they have experienced that […]*). Contrary to expectations, scientific refutation texts were shown to be more effective than anecdotal ones at dispelling beliefs in neuromyths. It should be noted, though, that beliefs in neuromyths were measured, not intentions to use neuromyth-derived teaching practices. Might anecdotal evidence, in particular personal experience, be more effective when it comes to dispelling intentions to use neuromyth-derived teaching practices?

Rousseau and Brabant-Beaulieu (2020) combined scientific refutation lectures with anecdotal evidence artificially created in the laboratory to dispel intentions to use neuromyth-derived teaching practices. Pre-service teachers were invited to participate in a “pedagogical activity” without being aware that the activity aimed at creating a personal anecdote unsupportive of the “learning styles” neuromyth. Participants were asked to memorize word pairs, the first half being accompanied by images and the other half by sounds. A cued-recall test followed, with the first word serving as the cue. Afterwards, participants were categorized either as more “Visual” or more “Auditory” learners, according to their score on learning style questionnaires. To underpin the scientific arguments made in the refutation lecture, anecdotal data from the personal activity, illustrated on graphs, were presented side by side with similar graphs from peer-reviewed journal articles. Expectedly, both sources of evidence converged to make a strong case against the “matching hypothesis.” From 100% before the intervention, intentions to use teaching practices derived from the VAK learning styles neuromyth declined to 60%. Although the drop was statistically significant, the majority of pre-service teachers clung to their initial intentions despite being confronted with converging scientific and anecdotal counterevidence.

In real life, it may be the case that anecdotal evidence is efficient because it confirms held beliefs (confirmation bias; Riener and Willingham, 2010). That anecdotal narratives used in refutation texts (Menz et al., 2021a) or anecdotal data from a personal experience (Rousseau and Brabant-Beaulieu, 2020) disconfirm—rather than confirm—held beliefs, could potentially explain why this kind of intervention is not efficient at dispelling beliefs in neuromyths or intentions to use neuromyth-derived teaching practices.

Given that intuitive/anecdotal evidence might contribute to consolidate false beliefs through a variety of cognitive biases (availability, familiarity, confirmation biases), some intervention approaches focus upon making educators more aware of the propensity of the human mind to rely on intuitive thinking at the expense of rational thinking. For example, McMahon et al. (2019) invited pre-service teachers to take part in an informal reproduction of the well-known “seductive allure of neuroscience” experiment (Weisberg et al., 2008). Although this was not the only component of their 90-min workshop intervention, once participants realized that simply adding superfluous, irrelevant neuroscience information to a claim can bias judgments toward it, endorsement of neuromyths, on a 7-point Likert scale (from 1-*Definitely false* to 7-*Definitely true*), went from upper ratings to mid-point ratings (uncertainty). Menz et al. (2021c) recently evaluated the efficiency of a short training session on cognitive biases at dispelling misconceptions, in a sample of pre-service teachers. The online training comprised definitions of common cognitive biases, along with concrete examples (e.g., confirmation bias: *People who deny human-caused climate changes only search for and read information regarding this topic that displays their attitude regarding climate changes*). In addition, participants generated their own example for each bias. Although this training did not lead to fewer endorsement of misconceptions, conclusions based on a single attempt should be taken with caution. Future attempts at making teachers more aware of their cognitive biases, by using a different training format and/or more extensive training sessions, might show to be beneficial. Others (e.g., Rousseau et al., 2018; Tardif, 2020) have suggested that critical thinking skills should be part of initial teacher training, in order for educators to develop a healthy skepticism helping them to better distinguish science from pseudoscience and to nurture more realistic expectations regarding what neuroscience can bring to education.

## Do Reduced Beliefs in Neuromyths Translate in the Adoption of More Evidence-Based Teaching Practices?

As previously stated, the impact of believing in neuromyths on educators' pedagogical choices is still unknown. Although teachers have been asked to report on whether or not (or to what extent) they incorporate neuromyths into their daily teaching practices (Blanchette Sarrasin et al., 2019; Hughes et al., 2020) and, if so, examples of ways they incorporate them (Hughes et al., 2020; Papadatou-Pastou et al., 2021), or to indicate their intention to use accredited and unaccredited teaching practices (Ruhaak and Cook, 2018), behavioral evidence is still dramatically lacking. For that reason, Torrijos-Muelas et al. (2021) called for research on neuromyths “to move into the classroom at every level” (p. 16). The same rationale holds for interventions to dispel beliefs in neuromyths or intentions to use neuromyth-derived teaching practices. As rightly stated by Newton and Salvi (2020) and by Lithander et al. (2021), behavioral evidence is needed to measure the impact of refutation-based interventions on educators' teaching practices.

A first step in that direction could be made by using a newly developed instrument. As an alternative^6^ to Dekker et al.'s 2012 standard, “true/false” single-statement neuromyth survey, Tovazzi et al. (2020) recently developed a *practice-oriented*, multi-option instrument called the *Neuroscience against Neuromyths Questionnaire* (NNQ). For each item, educators are first presented with a realistic classroom scenario (e.g., how to memorize that list), followed by four pedagogical choices. Some choices are based on prominent neuromyths, others are evidence-based, and others are distractors. The NNQ provides a way to assess whether educators' pedagogical choices are influenced by neuromyths. Tovazzi et al. (2020) administered both a standard survey adapted from Dekker et al. (2012), as well as the NNQ, to a sample of in-service teachers. Remarkably, participants were less likely to fall for neuromyths when tested with the multi-option instrument than with the standard one. This important finding suggests that “true/false” single-statement surveys, vulnerable to response biases, might possibly *overestimate* the prevalence of neuromyth beliefs in educational settings. Indeed, “true” answers to neuromyth single statements may not necessarily reflect educators' blind endorsement of “quick-fix,” misinformed teaching practices. On the contrary, presented with alternative, scientifically sound pedagogical options to deal with realistic classroom scenarios, teachers do not appear prone to endorse neuromyth-derived practices.

Some authors (e.g., Alferink, 2007; Grospietsch and Lins, 2021) advocate that as long as teachers are not being adequately provided with alternative, evidence-based teaching practices (e.g., Dunlosky et al., 2013; Agarwal and Roediger, 2018; Weinstein et al., 2018), pseudoscientific sources of knowledge will prosper and neuromyth-derived practices will prevail. However, dispelling neuromyths through refutation-based interventions, as well as dictating “good practices” through neuroscience-to-education translational efforts, both contribute to a one-way, authoritative dialog between scientists and educators. Although teachers are generally positive toward research and the use of neuroscience knowledge to inform their teaching (e.g., Pickering and Howard-Jones, 2007; Dubinsky, 2010; Rato et al., 2011; Serpati and Loughan, 2012; Guilbert et al., 2016; Papadatou-Pastou et al., 2017; Ching et al., 2020), frontal attacks on their daily teaching practices may result in widening, rather than narrowing, the science-practice gap. In an oft-cited call, Fischer et al. (2007) advocated a two-way, bidirectional dialog between scientists and educators. To foster such a dialog, some intervention approaches promote *non-prescriptive* ways to instill neuroscience in the classroom.

## Are Teacher Professional Development Workshops and Seminars on the Neuroscience of Learning Effective at Instilling Neuroscience in the Classroom?

Aside from research on surveying and dispelling neuromyths, a whole research area focuses on *non-prescriptive* ways to instill knowledge about the neuroscience of learning into teachers' practices. These innovative approaches, developed in the last 15 years, are mainly based on teacher professional development seminars and workshop: the “Brain Science on the Move” program (MacNabb et al., 2006), the “BrainU” summer workshops (Dubinsky, 2010; Roehrig et al., 2012; Dubinsky et al., 2013); “The Neuroscience of Learning and Memory” and other workshops (Dommett et al., 2011), “Learning and the Brain” conferences (Hook and Farah, 2013), and more recently a 36-h “Neuroscience for Educators” course (Schwartz et al., 2019; Chang et al., 2021). Rather than being trained on *researcher-initiated* neuroscience applications, teachers are granted agency on how to use acquired neuroscience knowledge in their practice, with the aim of promoting *teacher-initiated* applications:

Within a constructivist setting, teachers may make personal meaning by combining the neuroscience and their own insights to the relevant contexts of their classrooms (Chang et al., 2021, p. 2).

A key finding of these case studies is that attending seminars and workshops on the neuroscience of learning enriches teachers' pedagogical choices. Of critical note, changes in teaching practices are not only documented through interviews, but also through direct classroom behavioral observations.

## Summary, Research Gaps and Future Directions

Interventions to dispel neuromyths in educational settings have mainly focused on refutation texts/lectures. Considering only this intervention approach, many issues are still pending. First, refutation texts go from the “This statement is false” single line (Lithander et al., 2021) to elaborate texts about the origin of the neuromyth and neuroscientific counterevidence (Ferrero et al., 2020a,b), alone or in conjunction with personal reflections to foster a conceptual change (Grospietsch and Mayer, 2018). The key mechanism underlying the corrective effects is still largely unknown. Second, paradoxically, whereas a single-line refutation text brings enduring effects, lasting at least 1 month (Lithander et al., 2021), effects of elaborate refutation texts/lectures sometimes vanish within 30 days (Ferrero et al., 2020a,b), and sometimes endure up to 6 months (Menz et al., 2021c) and even 2 years (Kowalski and Taylor, 2017). The reasons for such discrepancies in outcomes is an open question. Third, whether corrective effects on neuromyth beliefs extend to teaching practices in the classroom is currently unknown. This lack of behavioral data constitutes a major limitation of current refutation-based intervention approaches. Fourth, the social desirability bias may represent the more important methodological pitfall. One way to possibly mitigate this bias would be to wait several weeks, rather than re-administering the neuromyth survey just after the participants have read the refutation texts. Fifth, because neuromyths have been shown to be maintained by intuitive thinking (Bensley et al., 2014; Newton and Miah, 2017; Blanchette Sarrasin et al., 2019; Menz et al., 2021b), refutation-based interventions, focused on rational thinking, may not be enough to dispel them. Innovative ways to debunk neuromyths by mitigating intuitive thinking through using anecdotal evidence (Rousseau and Brabant-Beaulieu, 2020; Menz et al., 2021a), as well as through training sessions on cognitive biases (McMahon et al., 2019; Menz et al., 2021c), deserve to be explored further.

With regards to neuroscience training as a “protective shield” against neuromyths, merely taking an undergrad course in the area (i.e., passive exposure) tends to be insufficient to reduce beliefs in neuromyths, even though lectures and textbooks comprise elements that contradict the neuromyths (Kowalski and Taylor, 2017; Grospietsch and Mayer, 2018; Im et al., 2018; but see Menz et al., 2021c, for an exception). Having taken several brain-related or neuroscience courses at the undergrad level provides educators with some, but “modest” protection against neuromyths (Macdonald et al., 2017). Unlike neuroscience training at the undergrad level, training in the context of teacher professional development (workshops, seminars) looks promising (e.g., Dubinsky, 2010; Hook and Farah, 2013; Chang et al., 2021). Although effects of attending these workshops/seminars on neuromyth beliefs are unknown, positive effects on teachers' pedagogical choices have been evidenced from direct classroom behavioral observations. This apparently opposite pattern of results (no beneficial effects of neuroscience training at the undergrad level vs. beneficial effects in the context of professional development) might possibly indicate that in-service teachers do not attend neuroscience training with the same “state of mind” as teacher students. While the latter population might be more grade-oriented, the former population might be more likely to establish links between the newly acquired knowledge and the way they dealt with generations of students over their career. Maybe in-service teachers “click” more to neuroscience training than prospective teachers because they can pinpoint exactly *how* each piece of information may fit within their own arsenal of pedagogical strategies to properly address recurrent learning challenges met in the classroom. This hypothesis deserves to be explored further. As well, because beliefs in neuromyths were not assessed, it would be much interesting to seek out if observed changes in teachers' pedagogical choices, following neuroscience workshops/seminars, translate in higher correct scores on Tovazzi et al.'s 2020 multi-option, practice-oriented NNQ instrument, in comparison to non-attendees holding the same level of teaching experience.

## Conclusion

The high prevalence of beliefs in neuromyths among educators did not decline over the past decade. Although there is yet no direct evidence that believing in these misconceptions hinders teaching practices, educational policies are already influenced by neuromyths, resulting in unreasonable spending of resources and money. Intervention approaches that focus on both activating rational thinking (i.e., refutation-based interventions) and mitigating intuitive thinking, as well as non-prescriptive approaches like teacher professional development workshops and seminars on the neuroscience of learning, are promising avenues to dispel beliefs in neuromyths and to instill evidence-based teaching practices in the classroom, respectively. Future research should seek to combine both avenues to draw a more complete picture of the neuromyth phenomenon in educational settings and to combat it, from misconceptions to misinformed teaching practices.

## Data Availability Statement

The original contributions presented in the study are included in the article/supplementary material, further inquiries can be directed to the corresponding author/s.

## Author Contributions

The author confirms being the sole contributor of this work and has approved it for publication.

## Funding

This work was supported by the Canada Foundation for Innovation John R. Evans Leaders Fund grant 18356.

## Conflict of Interest

The author declares that the research was conducted in the absence of any commercial or financial relationships that could be construed as a potential conflict of interest.

## Publisher's Note

All claims expressed in this article are solely those of the authors and do not necessarily represent those of their affiliated organizations, or those of the publisher, the editors and the reviewers. Any product that may be evaluated in this article, or claim that may be made by its manufacturer, is not guaranteed or endorsed by the publisher.

## Footnotes

^1^The weighted average was computed following Newton and Salvi (2020, p. 6).

^2^Note that in Carter et al., 2020, the neuromyth score was the percentage of neuromyth correct *identification* (rather than the percentage of endorsement), resulting in the report of a significantly positive correlation. This actually reflects a *negative* correlation between general neuroscience knowledge and beliefs in neuromyths.

^3^Note that in Kim and Sankey (2018), the neuromyth score was the percentage of neuromyth *correct identification* (rather than the percentage of endorsement), resulting in the report of a significant negative correlation. This actually reflects a *positive* correlation between general neuroscience knowledge and beliefs in neuromyths.

^4^According to Grospietsch and Lins (2021), a distinction between neuromyths and psychological misconceptions is unsustainable and counterproductive to the development of intervention approaches in the field.

^5^In conceptual change models, creating a *cognitive conflict* is assumed to favor the dismissal of an old (incorrect) conception and the adoption of a new (correct) one. However, recent research (Potvin et al., 2020) has shown that conceptual change is not an updating process: misconceptions are not “erased” or replaced by newly acquired, correct conceptions, but rather *coexist* with them. The misconception and the correct conception are in continuous competition in the mind, and one prevails over the other to provide guidance for behavioral choices. According to this “prevalence” conceptual change model (Potvin, 2013), the correct conception will prevail if proper *inhibition* is exerted on the misconception.

^6^Another alternative to surveys using a true/false, single-statement format is the *Test of Psychological Knowledge and Misconceptions* (TOPKAM; Bensley et al., 2014), which uses a two-alternative (A/B), forced choice format. The misconception statement is pitted against a scientifically valid option (see also Kowalski and Taylor, 2017).

## Dedication

This work is dedicated to the memory of Serge Bouchard (1947–2021), Canadian anthropologist.
